# The proteasomal inhibitor MG132 prevents muscular dystrophy in zebrafish

**DOI:** 10.1371/currents.RRN1286

**Published:** 2011-11-17

**Authors:** Steve J Winder, Leanne Lipscomb, Caroline Angela Parkin, Mikko Juusola

**Affiliations:** ^*^Department of Biomedical Science, University of Sheffield and ^‡^Centre for Developmental and Biomedical Genetics, Department of Biomedical Science, University of Sheffield

## Abstract

Using sapje zebrafish which lack dystrophin, we have assessed both the quantitation of muscle damage in dystrophic fish, and the efficacy of the proteasomal inhibitor MG132 in reducing the dystrophic symptoms. Fourier analysis of birefringence patterns in normal and dystrophic fish was found to be a simple and reliable quantitative measure of muscle damage. MG132, as in mdx mouse, was found to be effective in reducing muscle damage with an EC50 of 0.4µM. This study adds further to the utility of zebrafish as a model of choice for testing muscular dystrophy therapeutics.

## **Introduction**

The zebrafish *Danio rerio*, has rapidly been adopted as an organism of choice for all aspects of the drug discovery pipeline [[1],[2],[3]]. We have therefore developed a zebrafish medium-throughput platform as a test bed for therapeutic advancement for muscular dystrophies. The zebrafish system offers unique advantage for drug screening in a vertebrate model organism, and in particular muscular dystrophies are especially amenable due to their early, robust and readily recognisable phenotypes [[4],[5]]. Their small size, embryonic status, low cost and ease of drug delivery directly via the water make zebrafish a very attractive model for whole organism screening. Zebrafish show a typical vertebrate development pattern, and in the mutants, perturbation of muscle architecture and muscle function is readily observable even in the embryonic stages [[4],[5],[6]]. In addition, of the genes known to be mutated in human forms of muscular dystrophy, all but one are represented in the zebrafish genome and those investigated so far exhibit dystrophic phenotypes in zebrafish  [[7],[8]]. Although candidate compounds identified in fish would need to be validated in mammals before being taken on to human therapy, the low cost and speed of candidate drug screening, far outweighs any disadvantages. 

A recent screen from the Kunkel Group has also validated this approach and identified a number of compounds that appear effective in reducing dystrophic symptoms in zebrafish [9], in particular PDE5 inhibitors appear to be useful in this regard as they have also been shown to be effective in *mdx* mice [[10],[11]]. Previous studies from the Lisanti group and ourselves suggested that tyrosine phosphorylation of dystroglycan is an important mechanism for controlling the association of dystroglycan with its cellular binding partners dystrophin and utrophin, and also as a signal for degradation of dystroglycan [[12],[13],[14]]. The Lisanti group further demonstrated that inhibition of the proteasome was able to restore other dystrophin glycoprotein complex (DGC) components in both *mdx* mice that lack dystrophin and in explants of DMD patients [[15],[16]]. We have therefore chosen to examine the proteasomal inhibitor MG132 as a proof of principle in the zebrafish system comparing wildtype with dystrophic *sapje* larvae, which have a premature stop codon in the dystrophin gene, express no full-length dystrophin protein and exhibit a dystrophic phenotype [6].

## **Methods**

### Zebrafish

Heterozygous *sapje* ^t222a^ zebrafish [6] were maintained using standard procedures. Pairs of heterozygous *sapje* fish were mated, and embryos collected and raised at 28.5°C. Embryos 24 hours post fertilisation (hpf) were transferred in groups of 20 into 12-well plates containing 1ml of E3 media. Embryos were exposed to the proteasomal inhibitor MG132 (Calbiochem) at various concentrations from a DMSO stock diluted in E3 media, for 48 hours at 28.5°C. The final concentration of DMSO was 1% in both treated and control wells.   

### Analysis

At 3dpf embryos were anaesthetised in tricaine, and viewed between polarising filters on a dissecting microscope. The proportion of fish with an abnormal muscle birefringence, as determined by eye, was counted. For quantitative analysis, images were captured using a SPOT camera. Quantification of muscle damage was measured by taking a line scan from the 5th somite after the head along 1mm of the dorsal region of the somites in the direction of the tail. Line scans, *L*(*i*), in which *i* represents gray-scale intensities over the length of the sample, were subjected to standard Fourier analysis [17] and the resulting transforms, given as power spectra, were tested for significance by one-way ANOVA (Bonferroni-test) at selected spatial frequencies. In the Fourier analysis, the line scans (>2,000 points) were converted to mm-scale, divided into 50% overlapping stretches and windowed with a Blackman-Harris 4-term window [18] each giving seven to nine 250-points long samples. Thus, we obtained 7-9 spectral samples, which were averaged to improve the estimates of their power spectra, <|*L*(*f*)|^2^>, where | | denotes the norm, *f* spatial frequency and <> the average over the different stretches.

### Ethics Statement

No specific ethics approval under UK and EU guidelines was required for this study, as all zebrafish used were less than 5.2dpf, and are therefore not protected under the Animals (Scientific Procedures) Act. Embryos were obtained from adult zebrafish by a regulated procedure under the UK Home Office project licence number 40/3134. Adult zebrafish are maintained in UK Home Office approved facilities in the Medical Research Council Centre for Developmental and Biomedical Genetics aquaria at the University of Sheffield. 

## **Results and Discussion**

 Using birefringence as a marker of muscle damage, compared to wildtype larvae *sapje* zebrafish exhibit a typical mottled appearance (Figure 1AB), indicative of disruption to the muscle architecture and a dystrophic phenotype. The birefringence phenotype is not readily visible until around 72hpf, but in order to test compounds that may prevent the onset of muscular dystrophy, drugs must be added before fish can be phenotyped. *Sapje* is a recessive allele and behaves in a normal Mendelian manner. Consequently experimental procedures are carried out on a mixed population of fish comprising 25% wildtype, 50% heterozygote and 25% homozygote *sapje*. As can be seen from Figure 1B, the extreme dystrophic phenotype of the homozygote *sapje* fish is clearly visible. However potential treatments that may be beneficial, might not completely restore the normal muscle architecture and give a regular birefringence pattern as seen in Figure 1A. We therefore examined potential methods to quantify the extent of muscle damage (or recovery) in dystrophic *sapje* embryos. Simple quantification of the brightness of the birefringence was found to be unreliable for two reasons. Birefringence is very orientation dependent and if embryos are not aligned in precisely the same way, relative to each other, position dependent changes in birefringence result, which have nothing to do with changes in muscle structure. Furthermore, simple brightness over the whole fish cannot distinguish between a fish with some dark and some bright somites, such as in Figure 1B, or a fish with a low level of muscle damage giving a more even but generally reduced birefringence (data not shown). We therefore chose to use line scanning and Fourier analysis to more precisely quantify the muscle damage. Figures 1CD, represent line scans of the individual fish shown in Figures 1AB, with the yellow bar representing the position of the scan. Wildtype fish have a regular and even pattern of birefringence, with brighter peaks in the somite and darker troughs corresponding to the myotendinous junctions. *Sapje* fish however, show a very irregular pattern of peaks and troughs due to the disruption of the birefringence pattern induced by the lack of dystrophin (Figure 1D). Fourier analysis of multiple wildtype or *sapje* fish is shown in Figure 2. Compared to wildtype, which at 3dpf have a single well defined intensity frequency of 10/mm corresponding to the observable somite boundaries, *sapje* fish have a completely disordered frequency distribution, with major peaks at 4 and 10/mm but many other intervening frequencies representing the very broken birefringence pattern in these fish. Statistical analysis of these frequency plots reveals a clear statistical difference (p=0.029) comparing as few as 4 wildtype with 6 mutant fish. This analysis provides a straightforward quantification method to determine the extent of muscle damage in zebrafish.   

**Figure:**
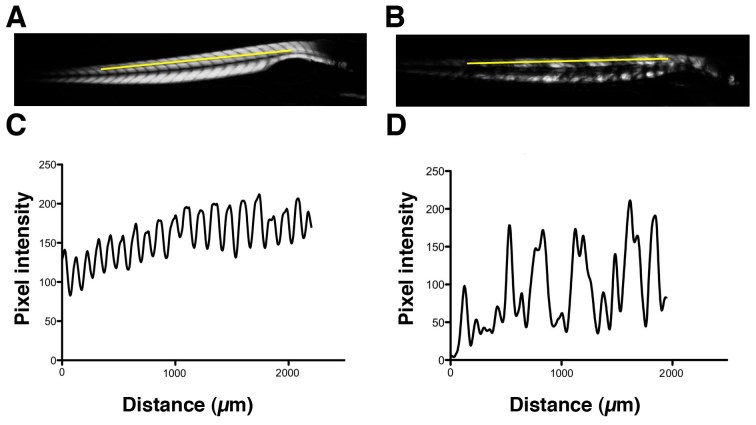
Figure 1. Birefringence images of wildtype (A) and *sapje* (B) zebrafish at 3dpf. Yellow bar shows the position of the line scan used to generate the intensity profiles shown in C and D for wildtype and *sapje* fish respectively.

**Figure:**
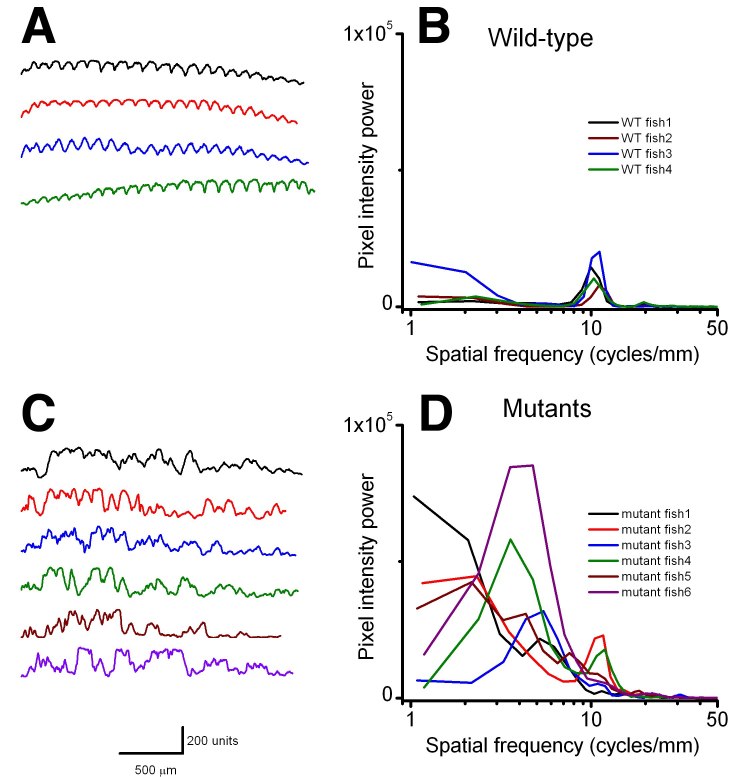
Figure 2. Line scans and corresponding Fourier analysis from wildtype (A,B) and 3dpf *sapje* (C,D). Left-hand plots (A,C) show the raw individual line scans for each fish used. Right-hand graphs (B,D) show the Fourier transforms of the data. Wildtype fish have a single frequency of 10/mm representing the actual number of somites per mm in these fish. *sapje* fish, whilst showing an underlying frequency of 10/mm, due to the disruption to the birefringence caused by the lack of dystrophin, also exhibit several other smaller frequency modes. As is clear to the eye, pixel power of wildtype and mutant fish populations are significantly different. At 4 cycles/mm, WT: 748 ± 536 and mutant: 36504 ± 26159 (mean ± SD, p = 0.029; one-tail ANOVA).

To validate further the *sapje* model we used the proteasomal inhibitor MG132, which has previously been shown to have beneficial effects in *mdx* mice [[19],[20]]. Treatment of *sapje* zebrafish with MG132 significantly reduces the number of dystrophic fish with an aberrant birefringence pattern from ~25% to ~15%, a rescue of approximately 40%. Titration of MG132 levels indicated a concentration-dependent restoration of birefringence in *sapje* zebrafish compared to DMSO vehicle alone. Expressed as percentage rescue of the 25% dystrophic population, the effect of MG132 reached a plateau at around 2µM with an EC50 of 0.4µM (Figure 3). This effective concentration range is an order of magnitude lower than doses reported to be effective in the hindlimb of *mdx* mice [20] or in explants from BMD and DMD patients [19]. This may however be attributable to the relative permeability of zebrafish embryos to water borne agents and therefore reflects the ease of delivery and effective dose achieved in the tissue, rather than any real difference in efficacy in fish as compared to mammals.

**Figure:**
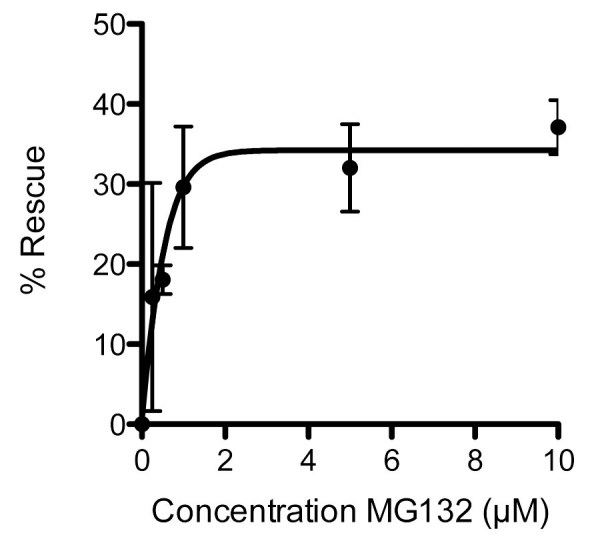
Figure 3. Dose response curve for the effect of MG132 in rescuing the dystrophic phenotype in *sapje* zebrafish. Each data point represents the percentage rescue of the dystrophic phenotype, taking the proportion of dystrophic fish in vehicle alone (1%DMSO: 0 µM MG132) as 0% rescue. Data are mean ± SEM of three independent experiments, following administration of MG132 or vehicle from 1dpf to 3dpf. EC50 = 0.4µM, maximal effective dose 2µM.

## **Summary**

This study adds further to the utility of zebrafish as a model of choice for testing muscular dystrophy therapeutics. In addition to the previous study validating the efficacy of PDE inhibitors [9] we can now add proteasomal inhibitors. Furthermore, zebrafish have also been demonstrated to be suitable for the testing of exon skipping strategies to treat muscular dystrophy[21], making them an invaluable part of the toolkit for the evaluation of a range of muscular dystrophy therapies. 

## **Acknowledgements**

We are grateful to all aquaria staff for fish husbandry.

## **Funding Information**

Work was funded by the Muscular Dystrophy Campaign and Medical Research Council.

## **Competing Interests**

The authors have declared that no competing interests exist.
